# PYRAMA: an open-source tool for advanced meta-analysis of genome wide association studies

**DOI:** 10.1093/bioinformatics/btag054

**Published:** 2026-02-02

**Authors:** Georgios A Manios, Sophia Nteli, Panagiota I Kontou, Pantelis G Bagos

**Affiliations:** Department of Computer Science and Biomedical Informatics, University of Thessaly, Lamia 35131, Greece; Department of Computer Science and Biomedical Informatics, University of Thessaly, Lamia 35131, Greece; Department of Mathematics, University of Thessaly, Lamia 35131, Greece; Department of Computer Science and Biomedical Informatics, University of Thessaly, Lamia 35131, Greece

## Abstract

**Motivation:**

Genome-wide association study (GWAS) meta-analysis tools are essential for integrating summary statistics across multiple cohorts, thereby increasing statistical power and validating genetic associations. Widely cited tools, such as METAL, PLINK, and GWAMA, have facilitated numerous significant discoveries in the field of GWAS. Nevertheless, these tools offer a limited set of meta-analysis methods and typically require users to have prior experience with command-line tools to be executed.

**Results:**

We present here PYRAMA, an open-source tool which is designed for meta-analysis of genome wide association studies. This work introduces an easy-to-use software package that includes several meta-analysis methods that are absent in similar software packages. PYRAMA is faster compared to other tools, supports robust methods for analysis and meta-analysis, fixed-effects, random-effects and Bayesian meta-analysis and it is currently the only tool that supports meta-analysis with imputation of summary statistics. It is available both as a standalone tool and as a freely available web server.

**Availability and implementation:**

https://github.com/pbagos/PYRAMA, https://doi.org/10.5281/zenodo.17830449.

## 1 Introduction

Various statistical methods are used in Genome Wide Association Studies (GWAS) aimed at identifying genetic variants associated with complex diseases (Ziegler *et al.* 2008, Uffelmann *et al.* 2021). Multiple key discoveries have emerged from the analysis of GWAS summary statistics (Kontou and Bagos 2024), with their availability enhancing accessibility to genetic data and advancing genomics. Meta-analysis, by combining multiple studies, increases statistical power for variant detection (Evangelou and Ioannidis 2013). Several software tools have been developed for conducting meta-analyses using GWAS summary statistics and prominent examples of such tools include PLINK (Purcell *et al.* 2007), METAL (Willer *et al.* 2010), and GWAMA (Mägi and Morris 2010). These tools have been extensively used in scientific literature and have played an important role in numerous significant genetic discoveries in this field. However, these tools perform standard meta-analysis methods using summary statistics as input, assuming the genetic model a priori (usually, the per allele model), or lack other important features. Therefore, there is a need for a tool in which the users will be able to perform GWAS meta-analysis and choose among several methods. Furthermore, performing summary statistics imputation is imperative to assign untyped loci in a study, thereby augmenting statistical power in the meta-analysis.

We present PYRAMA (Python tool for Robust Analysis and Meta-Analysis), an open-source package for analysis and meta-analysis of GWAS. PYRAMA is available as a standalone tool and a free web server, supporting fixed and random effects models, robust tests, Bayesian meta-analysis, summary statistics imputation using precomputed LD panels, and functional enrichment analysis.

## 2 Materials and methods

PYRAMA can be used to perform a broad range of tasks related to the meta-analysis of GWAS. It consists of the following components: Quality Control, Standard GWAS meta-analysis, Robust analysis and meta-analysis of GWAS, Bayesian meta-analysis, Meta-analysis with summary statistics imputation, and Functional Enrichment analysis.

A typical meta-analysis in PYRAMA can be conducted using standard summary statistics input files that provide effect size estimates (odds ratios for binary traits or beta coefficients for continuous traits), along with their corresponding standard errors. Furthermore, PYRAMA accepts input data involving both discrete (binary) and continuous phenotypes to proceed to an analysis or a meta-analysis. Before conducting an analysis or a meta-analysis, we recommend that users execute the quality control script that accompanies PYRAMA. This script performs essential preprocessing steps, including the removal of problematic rows from the input studies, verification of allele order (harmonization) and allele consistency across all datasets, and generation of a comprehensive quality control report. Additionally, it calculates the number of shared variants for each combination of studies, providing users with a clear overview of the variant overlap among the imported studies. Although there are several tools that are dedicated to perform extensive quality controls in GWAS summary statistics, such as EasyQC (Winkler *et al.* 2014), MungeSumstats (Murphy *et al.* 2021), and TidyGWAS (Harder *et al.* 2025), these typically perform many tasks and rely on external reference files becoming memory intensive to be executed when applied to large datasets. In contrast, the quality controls implemented in PYRAMA focus on common problems that can affect the meta-analysis, such as allele flips that lead to combining studies reporting associations in opposite directions for the same variant as if they provided concordant rather than conflicting evidence (Begum *et al.* 2012) or corrupted summary statistics. Similar simple preprocessing strategies are also implemented in GWAMA and METAL.

In most of the cases knowledge of the underlying genetic model of inheritance is not known, and various so-called robust methods can be utilized to achieve higher power (Freidlin *et al.* 2002, Bagos 2013). Dimou *et al.* introduced a STATA program named GWAR (Dimou *et al.* 2017), which implemented MIN2 (Burton *et al.* 2007), MAX (Zang *et al.* 2010), and MERT (Freidlin *et al.* 2002) robust tests to perform analyses and meta-analyses in GWAS. In addition to the aforementioned tests, the MinP, Cauchy, MCM, CMC (Chen 2022), and Stouffer’s method for combining correlated tests without weights (Strube 1985) combination tests were implemented in PYRAMA, offering a faster and equally precise alternative to MIN2 and MAX, while avoiding the computational burden of numerical integration (Nikolitsa *et al.* 2025).

In the standard GWAS meta-analysis function, both fixed-effect and random-effects models are supported using the inverse-variance weighting approach, as presented in Equations (1)–(4) of the Supplementary Material, available as supplementary data at *Bioinformatics* online. The most commonly used estimator for heterogeneity is the DerSimonian and Laird method of moments (DerSimonian and Laird 1986), but several others are supported by the tool (Cochran 1954a, Hedges 1983, Sidik and Jonkman 2005). Furthermore, when the input file adheres to the discrete or continuous phenotypes case, the Cochran Armitage Trend Test (Cochran 1954b, Armitage 1955), known as CATT can also be used as effect size, under all inheritance models (additive, dominant, or recessive).

Besides the standard frequentist approaches, Bayesian methods can be used in GWAS meta-analyses. In this work, an approximate Bayesian approach (Abrams and Sansó 1998), is implemented. Due to the simplicity of this approach, it is possible to make an estimation of the posterior by using first and second-order Taylor series expansions and obtain closed-form expressions. See the Supplementary Material, available as supplementary data at *Bioinformatics* online for more details of the method.

Imputation is widely used in GWAS to increase genomic coverage and enhance statistical power in meta-analyses (Li *et al.* 2009). With individual participant data (IPD), genotype imputation infers untyped variants from a reference panel, producing dense variant sets across cohorts. While accurate, it can be computationally intensive and requires individual data, which may not be available. To address these issues, many summary statistics imputation methods have been developed that offer equally accurate results with genotype imputation tools (Lee *et al.* 2013, Kwan *et al.* 2016, Rüeger *et al.* 2018, Julienne *et al.* 2019). However, imputation must be performed before meta-analysis, which can be challenging in practice. YAMAS (Meesters *et al.* 2012) was the only tool to combine summary statistics imputation and meta-analysis under a common framework, but it is no longer actively maintained.

Thus, to extend the functionalities of PYRAMA, we have incorporated the summary statistics imputation method presented in PRED-LD (Manios *et al.* 2025), a recent summary statistics imputation tool that was developed in our laboratory. PRED-LD uses precomputed LD statistics from HapMap (Gibbs *et al.* 2003), Pheno Scanner (Staley *et al.* 2016), and TOP-LD (Huang *et al.* 2022) as reference panels to impute summary statistics, given beta coefficients and standard errors. PRED-LD was found to be a more efficient method in comparison to other software that was available. More details about the method and its implementation can be found in the respective manuscript. The imputation procedure in PYRAMA is implemented seamlessly in a single step and the user does not need to perform any preprocessing steps.

While numerous tools exist for performing functional enrichment analysis, such as Enrichr (Kuleshov *et al.* 2016), PANTHER (Mi *et al.* 2021), or FUMA (Watanabe *et al.* 2017), in this work we opted to use g: Profiler (Raudvere *et al.* 2019), a widely used resource that provides easy programmatic integration into post-GWAS workflows. Within PYRAMA, g: Profiler is used to perform functional enrichment analysis with a given rsID list, produced as the result of the meta-analysis. The software returns information containing gene definitions, a list with statistically significant enriched GO terms, biological pathways, regulatory motifs in DNA, and phenotype ontologies with which these genes are highly enriched. Additionally, it offers the user the option to visualize results using a Manhattan plot. Of course, the output of PYRAMA is a simple list and hence the users can choose to perform enrichment with any other available tool.

## 3 Results

### 3.1 Comparison to other tools

We compared the features of PYRAMA (Fixed Effect Meta-analysis, Random Effects Meta-analysis, Robust Methods, Summary Statistics Imputation, Bayesian Meta-analysis, Multithreading meta-analysis execution, and Web tool implementation) with the features of the available standard GWAS meta-analysis tools. PYRAMA is the most comprehensive, supporting all seven features compared. GWAR, the predecessor of PYRAMA, offered robust methods and a web tool, while METAL, PLINK, and GWAMA primarily supported meta-analysis with fixed and/or random effects models. SMetABF (Sun *et al.* 2022) was the only software tool under comparison that supported both Bayesian meta-analysis and web tool availability. We should mention that PLINK is an efficient toolset that is capable of a wide range of GWAS operations, for both individual and summary data, and it is not a dedicated meta-analysis package. The full comparison of the available features of each software is presented in Table 1.

**Table 1:** Feature comparison of the available GWAS meta-analysis tools.^a^

Tool	FE MA	RE MA	Robust Methods	SSI	Bayesian MA	Multithreading MA	Web tool
PYRAMA (Python)	✓	✓	✓	✓	✓	✓	✓
GWAR (STATA)	–	–	✓	–	–	–	✓
METAL (C++)	✓	–	–	–	–	–	–
PLINK (C/C++)	✓	✓	–	–	–	–	–
GWAMA (C++)	✓	✓	–	–	–	–	–
SMetABF (R)	–	–	–	–	✓	–	✓

a The compared features are Fixed Effect Meta-analysis, Random Effects Meta-analysis, Robust Methods, Summary Statistics Imputation, Bayesian Meta-analysis, Multithreading meta-analysis execution and Web tool implementation.

We compared the execution times of PYRAMA, PLINK, METAL, and GWAMA using simulated datasets containing 100 000, 500 000, and 1 000 000 variants, respectively, within the framework of standard GWAS meta-analysis using the inverse-variance model. PYRAMA and PLINK showed the shortest execution times and produced both fixed-effect and random-effects results within a single run. In contrast, GWAMA requires the meta-analysis model (fixed or random effects) to be defined in advance, and METAL supports only fixed effect meta-analysis. This comparison revealed consistent performance trends over varying dataset sizes and study counts of 10, 15, 20, and 25 studies. PYRAMA demonstrated fast and scalable performance, showing only minor discrepancies in execution time in comparison to PLINK across all scenarios, when executed with a single thread. When executed with four threads, a feature only available in this tool, PYRAMA achieved an average speed increase of 1.81× compared to PLINK, performing at nearly twice the speed. Further details on the comparison of execution times are provided in the Supplementary Material, available as supplementary data at *Bioinformatics* online.

### 3.2 Use case scenario

As a use case scenario, we obtained summary statistics from three publicly available GWAS of Parkinson’s disease, as reported in a GWAS meta-analysis of Evangelou and coworkers (Evangelou *et al.* 2007). In particular, the Mayo Tier 1 and Mayo Tier 2 summary statistics were accessed directly from the supplementary materials of the respective publication (Maraganore *et al.* 2005). Meanwhile, the summary statistics from the NINDS study (Fung *et al.* 2006) were obtained through the dbGaP database (Tryka *et al.* 2014), with accession number phs000089.v1.p1/pha000003.1.

The NINDS dataset comprised discrete phenotype counts, thus enabling the application of robust tests to identify potentially significant variants that were not identified under the additive model. No variants reached the conventional genome wide significance level in the dataset using the per-allele odds ratio, as none possessed a *P*-value lower than the conventional $5\;\times \;10^{-8}$. We calculated the *P*-values using the MIN2 and MAX methods in these data, using CATT as effect size and compared them to those reported in the NINDS dataset (with the per-allele Odds Ratio). The analysis revealed that the MAX method identified one variant (rs10963676) as statistically significant, while the MIN2 method identified three variants (rs9952724, rs850084, and rs1504212), all under the recessive model of inheritance. The particular variants already had a low *P*-value but did not reach genomewide significance. As expected, they have been identified in the past using other more advanced statistical methods, but their biological role remains unclear (Tang *et al.* 2009). The detailed comparison is depicted in Fig. 2, available as supplementary data at *Bioinformatics* online.

To replicate the results of the GWAS meta-analysis conducted by Evangelou and coworkers, we carried out both fixed-effects and random-effects meta-analyses across all common variants present in the three datasets, in an effort to reproduce the findings summarized in Table 3 of their publication, which highlights the significant variants in both fixed effect and random effects model. In Fig. 3, available as supplementary data at *Bioinformatics* online, we present the overlap of variants for each study combination of the Parkinson’s Disease studies. It is important to note at this point that SNPs located on the X chromosome were not detected in the NINDS dataset and were therefore excluded from our replication meta-analysis. Similarly, it is of importance to note that the authors of the original publication used 0.05 as level of significance in their meta-analyses and concentrated their attention only on the SNPs that were common in the three studies (507 SNPs). Despite these limitations, of the 49 SNPs reported in Table 3, 46 demonstrated statistical significance at the conventional level of significance 0.05 in PYRAMA. This finding was consistent with the results presented in Table 3 of Evangelou *et al.* Furthermore, we performed both fixed effects and random effects meta-analysis using all variants; however, no variant reached genome-wide significance $\left(P<1\times 10^{-8}\right)$.

To validate the accuracy of the summary statistics imputation method implemented in PYRAMA, we removed significant variants reported in Table 3 of Evangelou *et al.* from the Mayo Tier 1, Tier 2, and NINDS studies, then ran imputation with default settings (R2 threshold of 0.5, no MAF threshold, combined LD sources as reference panel). In Mayo Tier 1, removing variants, imputing, and meta-analyzing reproduced the same 46 significant variants (*P* < 0.05) as the standard meta-analysis (see Fig. 4, available as supplementary data at *Bioinformatics* online).

Finally, we assessed whether imputation boosts meta-analysis power by imputing variants missing in one or two studies. This increased variant coverage and, using random effects meta-analysis, identified seven genome-wide significant variants (rs3902057, rs271255, rs6968845, rs7852712, rs7481483, rs12323571, rs8058629) listed in Table 2, available as supplementary data at *Bioinformatics* online that could not be detected otherwise. Functional enrichment with g: Profiler revealed significant terms highlighting enrichment in neurodevelopment, transcriptional regulation, and cellular responses to environmental stimuli. Notably, these include variants of CRB1 that showed relevance due to its involvement in developmental and regulatory functions, of ELP4 that has previously been associated with neurodevelopmental disorders, of ELP4 known for its relevance in neurological conditions, and of RBFOX1 whose disruptions have been implicated in various neurological disorders, including epilepsy, autism spectrum disorder, and schizophrenia (more details and interpretation of the results in the Supplementary Material, available as supplementary data at *Bioinformatics* online).

## 4 Conclusion

We presented PYRAMA, a comprehensive and fast tool, addressing many limitations of the traditional GWAS meta-analysis software packages. To our knowledge, PYRAMA is the only active tool supporting both summary statistics imputation and meta-analysis. This feature can impute untyped loci and reveal associations otherwise missed, as shown in a Parkinson’s Disease meta-analysis where several SNPs reached genome-wide significance. It supports single and multi-threaded execution, outperforming comparable software in speed, making it suitable for large-scale studies. PYRAMA, is freely available in a public GitHub repository at: https://github.com/pbagos/PYRAMA and the web version of PYRAMA is available at: https://compgen.dib.uth.gr/PYRAMA. It is important to note that the web version of PYRAMA does not support the summary statistics imputation feature, to ensure that the meta-analysis will not be computationally intensive. PYRAMA requires summary genotype counts or summary statistics, and thus privacy is not a concern. Computational methods for GWAS and meta-analysis are active areas of research in our lab, and thus PYRAMA will be regularly updated with new features (i.e. meta-analysis methods, other random effects estimators, more LD reference panels, interactive visualizations, and more).

## Supplementary Material

btag054_Supplementary_Data

## Data Availability

All data generated or analyzed during this study, as well as the associated code, are available in the GitHub at https://github.com/pbagos/PYRAMA.
