# Supplementary material for: PYRAMA: an open-source tool for advanced meta-analysis of genome wide association studies
Source: Bioinformatics. 2026 Feb 2;42(2):btag054. doi: 10.1093/bioinformatics/btag054 (PMC12925247; doi:10.1093/bioinformatics/btag054)
Supplement: btag054_Supplementary_Data [file btag054_supplementary_data.docx]

**Supplementary file – PYRAMA: An open-source tool for advanced meta-analysis of genome wide association studies**

Table of Contents

[Supplementary Notes 2](#_Toc215571499)

[Standard GWAS Meta-analysis 2](#_Toc215571500)

[Bayesian meta-analysis 2](#_Toc215571501)

[Execution times 3](#_Toc215571502)

[Post-imputation meta-analysis results - Enrichment analysis 4](#_Toc215571503)

[Supplementary Figures and Tables 5](#_Toc215571504)

[Supplementary Figure 1 - Execution time for each tool 5](#_Toc215571505)

[Supplementary Figure 2 - Comparison of MAX and MIN2 robust methods under the Cochran-Armitage trend test (CATT) in the NINDS study 6](#_Toc215571506)

[Supplementary Figure 3 - Venn diagram for the overlapping variants of Parkinson’s disease meta-analysis 7](#_Toc215571507)

[Supplementary Figure 4 - Comparison of published and post imputation Z-scores with imputation in one cohort each time 8](#_Toc215571508)

[Supplementary Table 1 - Post imputation meta-analysis results 9](#_Toc215571509)

[References 9](#_Toc215571510)

# Supplementary Notes

## Standard GWAS Meta-analysis

In the standard GWAS meta-analysis function, both fixed-effect and random-effects models are supported using the inverse-variance weighting approach, as presented in equations (1)-(4),

$y_{i} \sim N (\theta_{i}, s_{i}^{2})$ (1)

$\theta_{i} \sim N (\mu,\tau^{2})$ (2)

$w_{i}^{*}=\frac{1}{s_{i}^{2}+\tau^{2}}$ (3)

$\hat{\mu}_{w}=\frac{\sum_{i=1}^{k} \hat{w}_{i}^{*}y_{i}}{\sum_{i=1}^{k} \hat{w}_{i}^{*}}$ (4)

where $y_{i}$ is defined as each study’s effect size, $s_{i}^{2}$ is the estimated variance of the true intervention effect of each study $i =1,\ldots,k$. $\theta_{i}$ refers to the effect of study $i$ and $\tau^{2}$ is the between-studies variance (heterogeneity). When $\tau^{2}$ =0 all studies estimate a common effect size, and the fixed effects model is recovered. The inverse variance weight is defined in equation (3) and in equation (4) the weighted average estimate $\hat{\mu}_{w}$ of the overall effect is calculated.

## Bayesian meta-analysis

Additional to the random effects model, we need to transform equation (1) to $y_{i}\sim N (\theta_{i}, s_{i}^{2}/n_{i})$. Given that we use Bayesian inference, it is necessary to assign a prior probability distribution to the parameter of interest. In this case, we use a gamma or an inverse-gamma ($IG$) prior distribution with two known parameters: $a$ and $b$, referred to as the shape and scale (or rate) parameters of the distribution. To define the statistical model of this approach, we use equations (5) and (6) in addition to equations to the random effects model equations as shown below,

$n_{i}={(n}_{1i}\times n_{2i})/{(n}_{1i}+n_{2i})$ (5)

$\tau^{2}\sim IG\left( \alpha,b \right)$ or $\tau^{2}\sim Gamma\left( \alpha,b \right)$ (6)

where $n_{1i}$ and $n_{2i}$ are denoted as the number of participants in the two groups (cases and controls) in the $i$th study. In case of continuous outcomes, the total sample size is used. The variance of the posterior distribution for the population parameter $\mu$ is expressed as:

$V\left( \mu\mid y,s^{2},n \right)\approx\frac{2\left( 1+bRSS/2 \right)}{bk\left( 2\alpha+k-3 \right)}$ (7)

The posterior expectation and the posterior variance of the between study variability $\tau^{2}$ are defined in equations (8) and (9), respectively.

$E\left( \tau^{2} \mid y,s^{2},n \right)\approx\frac{2\left( 1+bRSS_{B}/2 \right)}{b\left( 2\alpha+k-3 \right)}$ (8)

$V\left( \tau^{2} \mid y,s^{2},n \right)\approx\frac{8\left( 1+bRSS_{B}/2 \right)^{2}}{b^{2}\left( 2\alpha+k-3 \right)^{2}\left( k+2\alpha-5 \right)}$ (9)

And last, the posterior expectation for the population parameter $\mu$ is given by,

$$E\left( \mu\mid y,s^{2},n \right)\approx$$

$\frac{\bar{y}-\frac{b\left( k+2\alpha-1 \right)}{2\left( 1+bRSS_{B}/2 \right)}\sum_{i=1}^{k} \frac{n_{i}s_{i}^{2}}{n_{i}-3}\left( \frac{\bar{y}\left( k-3 \right)+y_{i}-\frac{\left( \bar{y^{2}}\left( \bar{y}-y_{i} \right)+y_{i}^{2} \right)\left( k+2\alpha+1 \right)b}{2\left( 1+bRSS_{B}/2 \right)}}{k} \right)}{1-\frac{b\left( k+2\alpha-1 \right)}{2\left( 1+bRSS_{B}/2 \right)}\sum_{i=1}^{k} \frac{n_{i}s_{i}^{2}}{n_{i}-3}\left( \frac{k-1}{k}\cdot\frac{\left( \bar{y}\left( \bar{y}-y_{i} \right)+y_{i}^{2} \right)\left( k+2\alpha+1 \right)b}{2\left( 1+bRSS_{B}/2 \right)} \right)}$ (10)

where ${RSS}_{B}$ is defined as ${RSS}_{B}=\sum y_{i}^{2}-k\bar{y}^{2}$ and $k$ is the number of studies that participate in meta-analysis, in all the above equations.

## Execution times

We compared the execution times of PYRAMA, PLINK (Purcell *et al.*, 2007), METAL (Willer *et al.*, 2010), and GWAMA (Mägi and Morris, 2010) using simulated datasets containing 100,000, 500,000 and 1,000,000 variants, respectively, within the framework of standard GWAS meta-analysis employing the inverse-variance model. Among all tools, PYRAMA and PLINK were the fastest, both producing fixed-effect and random-effects meta-analysis results simultaneously within a single run and recording the fastest execution times. In contrast, GWAMA requires the meta-analysis model (fixed or random effects) to be defined in advance, and METAL supports only fixed effect meta-analysis. This comparison revealed consistent performance trends over varying dataset sizes and study counts of 10, 15, 20 and 25 studies. PYRAMA demonstrated fast and scalable performance, showing only minor discrepancies in execution time in comparison to PLINK across all scenarios, when executed with a single thread. When executed with four threads, a feature only available in this tool, PYRAMA achieved an average speed increase of 1.81× compared to PLINK, performing at nearly twice the speed. METAL had the slowest performance across all scenarios, with longer execution times compared to the other tools (almost two times slower from the single thread execution of PYRAMA) and GWAMA came third in all evaluated scenarios. Overall, PYRAMA offers a balance of speed and scalability, making it an efficient choice for large-scale meta-analyses, as shown in Supplementary Figure 1. All the comparisons were conducted on a standard personal computer equipped with an Intel i3-1115G4 processor operating at a base frequency of 3.00 GHz, supported by 8 GB of RAM.

## Post-imputation meta-analysis results - Enrichment analysis

Enrichment analysis conducted after the post-imputation meta-analysis identified seven variants that reached genome-wide significance $(p < 1 \times{10}^{-8})$. Notably, rs3902057 in the CRB1 gene showed particular relevance due to its involvement in developmental and regulatory functions. Specifically, it was enriched in the Hippo signaling pathway (KEGG), previously implicated in Parkinson’s disease pathogenesis (Wei *et al.*, 2023), and multiple Gene Ontology terms related to organ development and morphogenesis, supporting its potential role in cell growth and tissue organization. The variant rs7481483 located within ELP4 also draws special attention. ELP4 encodes a subunit of the elongator acetyltransferase complex, essential for transcription elongation and translation processes. Dysregulation of ELP4 has previously been associated with neurodevelopmental disorders, reinforcing its relevance in neurological conditions (Addis *et al.*, 2015). Additionally, variant rs12323571 located in CRIP1, which encodes cysteine-rich protein 1, is noteworthy. CRIP1 functions primarily as a zinc-binding protein and plays a role in oxidative stress response and cell survival pathways (Ye *et al.*, 2025). Lastly, the variant rs8058629 within RBFOX1 also demonstrated high significance. RBFOX1 encodes an RNA-binding protein essential for neuronal development, synaptic plasticity, and RNA splicing. Disruptions of RBFOX1 have been implicated in various neurological disorders, including epilepsy, autism spectrum disorder, and schizophrenia, further highlighting its extensive role in neurological function and potential involvement in neurodegeneration (Vuong *et al.*, 2018). Importantly, neither rs271255 (located on chromosome 5) nor rs6968845 (mapped to the gene HRAT17 on chromosome 7) have prior associations reported in the GWAS Catalog or existing literature. Their absence from known variant databases suggests that these represent novel loci identified through our meta-analysis.

# Supplementary Figures and Tables

## Supplementary Figure 1 - Execution time for each tool


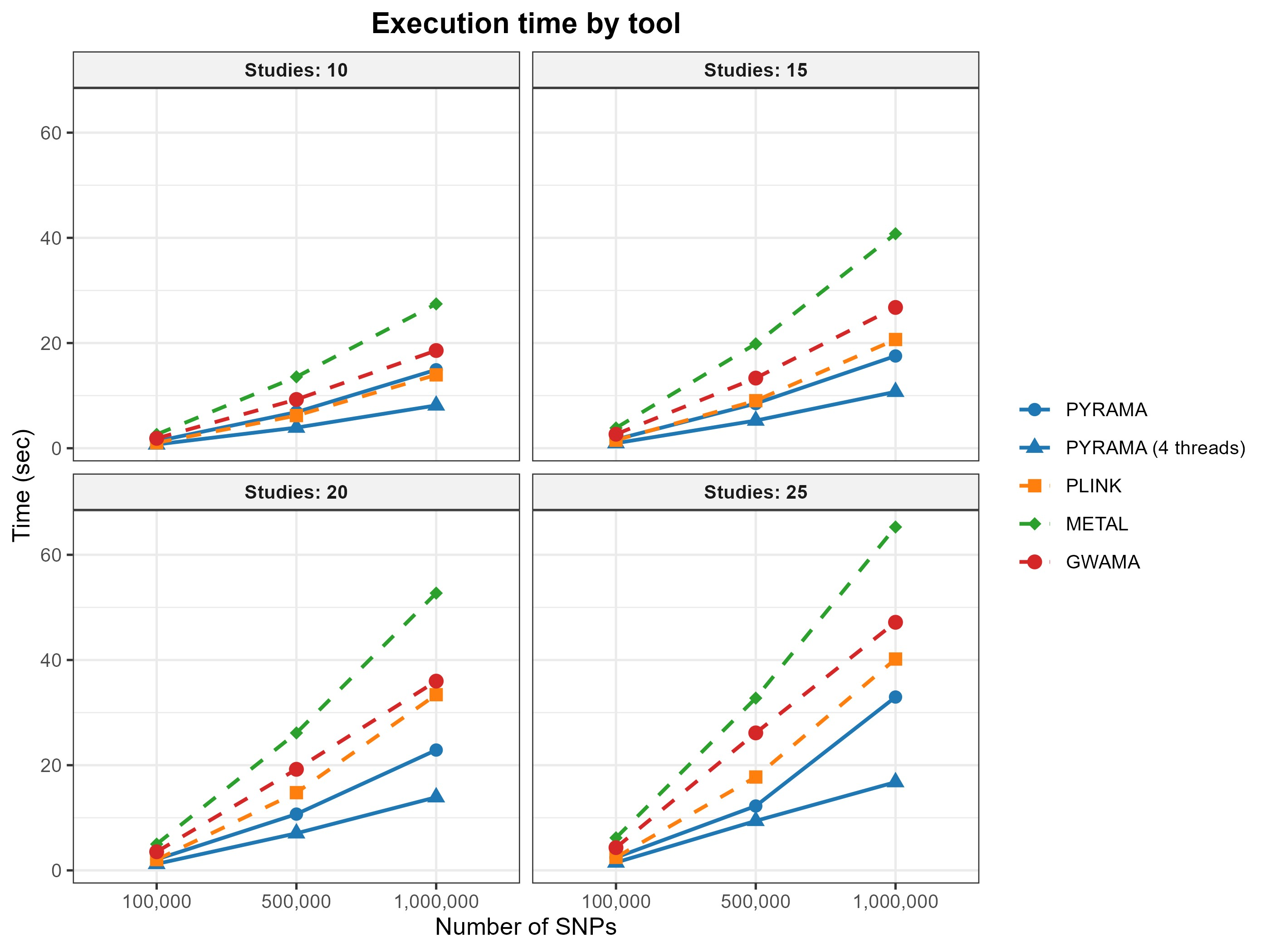
Each panel shows execution time (in seconds) as a function of the number of unique meta-analyses for the simulated datasets with 10, 15, 20, and 25 studies, respectively. PYRAMA consistently achieves the fastest execution times across all test cases, wheth-er running on a single thread or four threads. PLINK comes second in overall performance, while GWAMA and METAL require larger execution runtimes as both the number of unique meta-analyses and the number of studies increase.

## Supplementary Figure 2 - Comparison of MAX and MIN2 robust methods under the Cochran-Armitage trend test (CATT) in the NINDS study


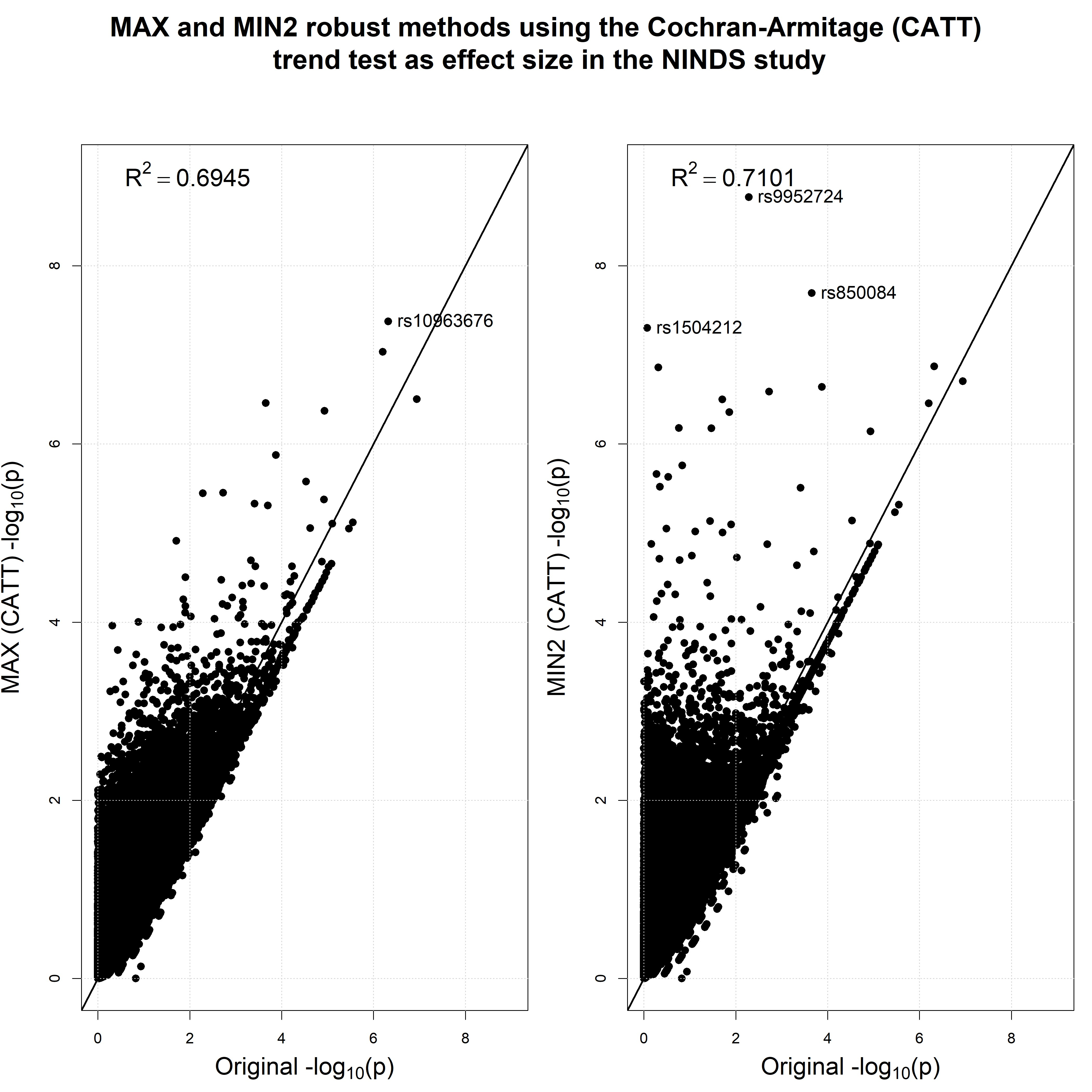
 The figure shows the relationship between the original –$log₁₀$($p$) values of the NINDS study and those obtained using the MAX (left panel) and MIN2 (right panel) robust tests. The variants that reached genome-wide significance ($p < 5 \times{10}^{-8}$) under each robust test analysis are highlighted in the figure. Note the various points above the diagonal line, indicating SNPs for which the robust method yields a smaller p-value compared to the per-allele Odds Ratio (most correspond to variants with a recessive model of inheritance).

## Supplementary Figure 3 - Venn diagram for the overlapping variants of Parkinson’s disease meta-analysis


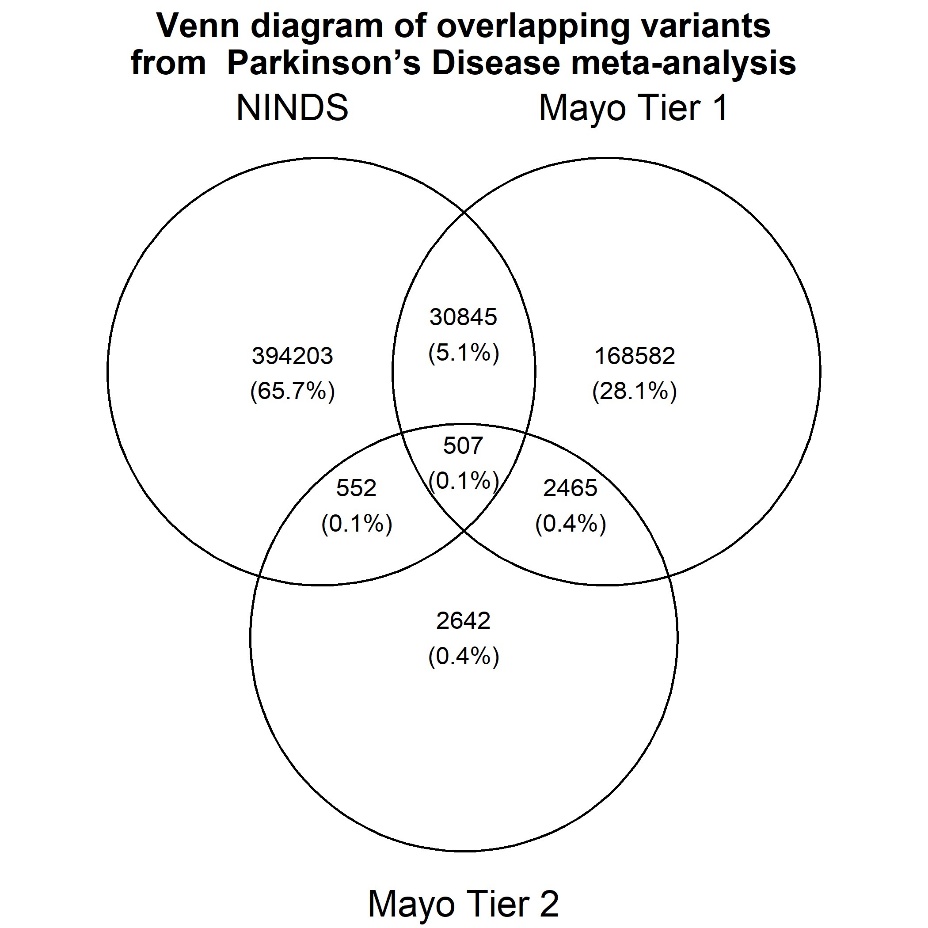
The overlap of variants from the Parkinson’s Disease meta-analysis of Evangelou et al. is displayed across the Mayo Tier 1, Mayo Tier 2, and NINDS studies. Most variants are unique to the NINDS study, while 507 variants are shared across all three datasets.

## Supplementary Figure 4 - Comparison of published and post imputation Z-scores with imputation in one cohort each time


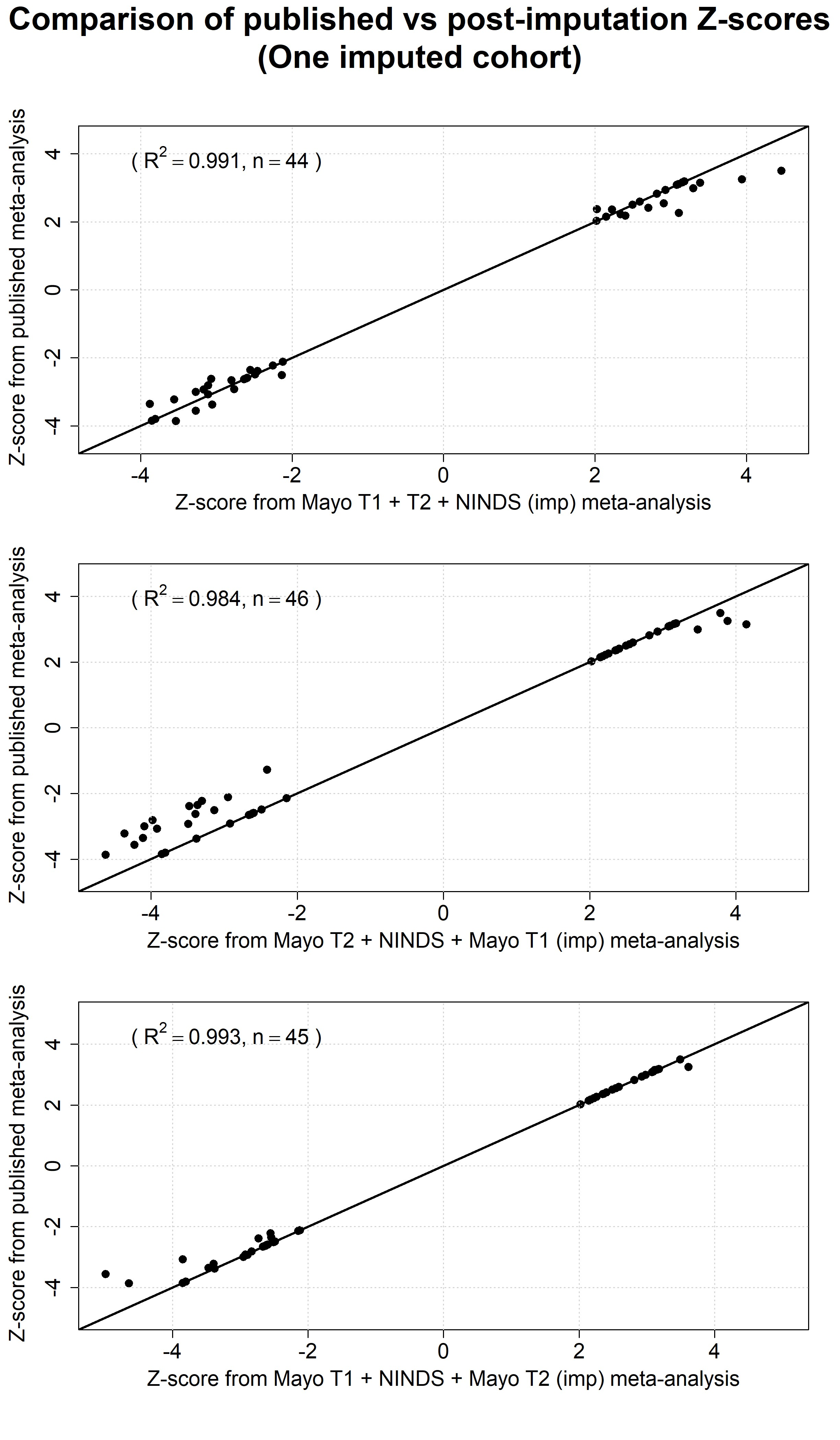
After removing the statistically significant variants of the initial meta-analysis scenario from the Mayo Tier 1 study, we performed imputation followed by a meta-analysis. We observed again the same 46 variants with the standard meta-analysis case as statistically significant, under the same level of significance $(p<0.05)$

## Supplementary Table 1 - Post imputation meta-analysis results

The imputation focused on the missing variants of Mayo Tier 1, Mayo Tier 2 and NINDS studies. After the imputation we performed meta-analysis, and we identified seven variants that reached genome-wide significance $(p < 1 \times{10}^{-8})$ under the random effects model. The "N" column denotes the number of studies in which each variant could be imputed and thus contribute to the meta-analysis, and the second number the total number of studies participating in the meta-analysis of each SNP. All variants were observed only once across the original datasets, prior to imputation.

| SNP | CHR | Gene Name | N | P-value  (RE) | Pooled  Standard  Error | Pooled  Effect size  (log (OR)) |
| --- | --- | --- | --- | --- | --- | --- |
| rs3902057 | 1 | CRB1 | 1/2 | 1.26e-17 | 0.062 | 0.536 |
| rs271255 | 5 | - | 1/2 | 5.01e-13 | 0.235 | 1.700 |
| rs6968845 | 7 | HRAT17 | 1/2 | 3.33e-09 | 0.095 | 0.563 |
| rs7852712 | 9 | LOC105375999 | 1/2 | 6.24e-27 | 0.085 | -0.922 |
| rs7481483 | 11 | [ELP4](https://www.genecards.org/cgi-bin/carddisp.pl?gene=ELP4&keywords=rs7481483) | 2/3 | 1.00e-300 | 0.009 | -0.688 |
| rs12323571 | 14 | CRIP1 | 1/2 | 5.45e-09 | 0.130 | -0.756 |
| rs8058629 | 16 | RBFOX1 | 1/2 | 6.73e-112 | 0.036 | 0.813 |

# References

Addis,L. *et al.* (2015) Microdeletions of ELP4 are associated with language impairment, autism spectrum disorder, and mental retardation. *Hum Mutat*, **36**, 842–850.

Mägi,R. and Morris,A.P. (2010) GWAMA: software for genome-wide association meta-analysis. *BMC Bioinformatics*, **11**, 1–6.

Purcell,S. *et al.* (2007) PLINK: a tool set for whole-genome association and population-based      linkage analyses. *Am. J. Hum. Genet.*, **81**, 559–575.

Vuong,C.K. *et al.* (2018) Rbfox1 regulates synaptic transmission through the inhibitory neuron-specific vSNARE Vamp1. *Neuron*, **98**, 127–141.

Wei,X. *et al.* (2023) An update on the role of Hippo signaling pathway in ischemia-associated central nervous system diseases. *Biomedicine & Pharmacotherapy*, **162**, 114619.

Willer,C.J. *et al.* (2010) METAL: fast and efficient meta-analysis of genomewide association scans. *Bioinformatics*, **26**, 2190–2191.

Ye,X. *et al.* (2025) Cysteine-rich intestinal protein family: structural overview, functional diversity, and roles in human disease. *Cell Death Discov*, **11**, 114.
